# Effective Extraction of Palmatine and Berberine from *Coptis chinensis* by Deep Eutectic Solvents-Based Ultrasound-Assisted Extraction

**DOI:** 10.1155/2021/9970338

**Published:** 2021-08-13

**Authors:** Lijing Li, Dong Zhang, Yuejie Wang, Fangxin Liu, Yang Xu, Huiwei Bao

**Affiliations:** ^1^College of Pharmacy, Changchun University of Chinese Medicine, Changchun 130117, China; ^2^College of Pharmacy, Baicheng Medical College, Baicheng 137000, China

## Abstract

The effective components of *Coptis chinensis* were extracted by ultrasound-assisted technology, and the contents of palmatine and berberine were used as indexes by using *Coptis chinensis* as raw material and eutectic solvent as extractant. In addition, the effects of hydrogen bond donor type, molar ratio of hydrogen bond donor to acceptor, material-liquid ratio, water content of eutectic solvent system, sonication time, power, and ultrasonic temperature on the extraction rate of palmatine and berberine were studied. The optimum extraction technology of palmatine and berberine from *Coptis chinensis* was determined by single-factor experiment and response surface optimization test. As a result, it showed that the eutectic solvent system was constructed with choline chloride as hydrogen bond acceptor and phenol as hydrogen bond donor, with a molar ratio of 1 : 3. In addition, water content of the eutectic solvent system was 30%, ratio of material to liquid was 30 g/mL, ultrasonic time was 30 min, ultrasonic power was 200 W, and ultrasonic temperature was 60°C. At this time, the contents of palmatine and berberine in *Coptis chinensis* were 16.7145 mg/g and 57.4013 mg/g, respectively, which were predicted to be the same as the value, and the extraction effect was better than that of traditional extraction solvent method.

## 1. Introduction

As the dry rhizome of *Coptis* c*hinensis*, *C*. *deltoidea* C. Y. Chenget Hsiao, and C. teeta Wall of Ranunculaceae, *Coptis* c*hinensis* are commonly known as Weilian, Yalian, Yunlian [1], bitter in taste and cold-natured [2], which can rise or fall [3]. Besides, it belongs to the heart, liver, stomach, and large intestine meridians, with the effects of clearing heat, dryness, and dampness, purging fire, and detoxification [1, 4]. In addition, *Coptis chinensis* was first published in the Classic of Shennong Materia Medica and was listed as a top-grade medicine [5]: “the smell is bitter, cold, and nontoxic.” The chemical constituents of *Coptis chinensis* include alkaloids, lignans, flavonoids, and acidic components [6]. The main active components are alkaloids [7], of which berberine is the main component. In addition, the contents of alkaloids such as palmatine, jatrorrhizine, and magnoflorine are high [8].

As first reported by Abbott et al. in 2003 [9], deep eutectic solvents (DES) are a eutectic mixture composed of hydrogen-bonded acceptors and hydrogen-bonded donors of solid halide salts with a certain stoichiometric ratio [10]. It is usually composed of two or more components, and its melting point is significantly lower than that of each component [11]. It has the advantages of simple preparation, low cost, and no need for complex purification. In addition, it is also widely used in electrochemistry, preparation of nanomaterials, catalytic reaction, separation process, and preparation of functional materials, among others [12–15]. It is only necessary to mix the hydrogen bond donor and hydrogen bond acceptor at a certain molar ratio in the preparation of DESs. In addition, heat and stir at a certain temperature until a uniform liquid is formed [16]. As extractant, eutectic solvent is often combined with ultrasonic-assisted extraction [17], microwave-assisted extraction [18], hollow fiber extraction [19], and so on. Eutectic solvents mainly have the following advantages in extraction [20]: ① the eutectic solvents synthesized by different components have different polarity, thus improving the extraction efficiency of different polarity analytes. ② Eutectic solvents have the advantages of easily available raw materials, low toxicity, biodegradability, no pollution to the environment [21], easy regeneration, and so on, compared with the traditional methanol extractant.

In this study, we performed the isolation and extraction method of palmatine and berberine from *Coptis chinensis* based on the ultrasound-assisted eutectic solvent. Besides, the effects of different DESs and other extraction factors on the extraction of effective components of *Coptis chinensis* were investigated through single-factor experiment. In addition, we combined with response surface method to optimize the extraction process of effective components of *Coptis chinensis* and compared with traditional water extraction and alcohol extraction. This study is committed to providing a greener, efficient, economical, and environmentally friendly new method for the extraction of alkaloids from *Coptis chinensis.* It can better promote the in-depth development of *Coptis chinensis* and provide data support for the development of green chemistry and the research of natural products of traditional Chinese medicine.

## 2. Experiment

### 2.1. Instruments and Materials

Firstly, the chromatographic analysis was carried out by using a high-performance liquid chromatography system (HPLC-Huapu S3000 provided with a four-element low-pressure stirring pump, automatic injector, diode array detector model 1100, and chemical workstation). In addition, a JP-300G ultrasonic cleaner was purchased from Wuhan Jiapeng Electronics Co., Ltd. Moreover, QL-901 Vortex instrument was purchased from Haimen Qilinbell Instrument Manufacturing Co., Ltd. Besides, TGL-16 freezing centrifuge was purchased from Hunan Xiangyi Laboratory Instrument Development Co., Ltd.

In addition, the *Coptis chinensis* was produced in Sichuan Province, China, purchased from Jilin Pharmaceutical Store (Changchun); these samples meet the requirements of *Coptis chinensis* in Chinese Pharmacopoeia (2020 edition). Berberine hydrochloride (≥85.9%) and palmatine hydrochloride (≥85.7%) standards were used for HPLC determinations which were purchased from China Food and Drug Identification Institute. Chromatographic grade methanol and acetonitrile were purchased from Fisher Company of the United States. All reagents were of analytical grade. Phosphoric acid was purchased from Tianjin Guangfu Technology Development Co., Ltd., and ultrapure water was purchased from Hangzhou Wa Co., Ltd. Choline chloride was purchased from Shanghai Zhanyun Chemical Co., Ltd. Maltose, malic acid, lactic acid, fructose, xylitol, and citric acid were purchased from Zhengzhou Kangyuan Chemical Products Co., Ltd. Phenol and acetic acid were purchased from Xilong Science Co., Ltd. Glycerin was purchased from Guangzhou Miya Cosmetics Co., Ltd. Propylene glycol was purchased from Tianjin Zhonghe Shengtai Chemical Co., Ltd. Urea was purchased from Tianjin Ruiji Chemical Co., Ltd.

### 2.2. HPLC Experimental Conditions

The chromatographic column was an Alltima^TM^ C_18_ column (250 mm × 4.6 mm, 5 *μ*m). Mobile phase included acetonitrile, 0.1%, phosphoric acid aqueous solution (30:70, v:v). The selected wavelength was 345 nm, the injection volume was 20 *μ*L, the flow rate was 1.0 mL/min, and the column temperature was 30°C.

### 2.3. Reference Solution

The reference substance of palmatine and berberine was precisely weighed and dissolved with methanol to obtain a mixed reference solution containing 0.572 mg/mL palmatine and 1.140 mg/mL berberine. The solution was precisely absorbed and diluted 1 mL 1 : 10 with methanol and filtered with a 0.22 *μ*m pore-size filter. In addition, the reference solution was obtained for further HPLC analysis.

### 2.4. Preparation of DESs

The hydrogen bond donor and hydrogen bond acceptor were mixed according to the molar ratio of Table 1. Besides, it was heated in a water bath at 80∼100°C and stirred well until a clear, transparent, and slightly viscous liquid solvent was obtained. For the selection of hydrogen bond receptors, choline chloride was selected, which was the mainstream and most widely used one in the market. In addition, for the selection of hydrogen bond donors, 11 kinds of maltose, malic acid, lactic acid, fructose, phenol, glycerol, propylene glycol, xylitol, urea, acetic acid, and citric acid were selected according to the principles of cost availability, safety, and controllability of raw materials.

### 2.5. Palmatine and Berberine Extraction from *Coptis chinensis* by DESs

50 mg *Coptis chinensis* was extracted (the powder passed through a 100-mesh sieve) and was added to eutectic solvent according to the ratio of material to liquid at 1 : 30 (g/mL). Besides, it was placed in 60°C warm bath for 5 min and was vortex oscillated for 5 min, and then ultrasonic extraction of 30 min (200 W) at 60°C was conducted and it was vortex oscillated for 1 min and centrifugation of 3000 rpm/min for 5 min was conducted.

### 2.6. Response Surface Test Design

Critical variables in the extraction process of *Coptis chinensis* were investigated, including DESs type, molar ratio, water content, material-liquid ratio, sonication time, ultrasonic temperature, and ultrasonic power. Several factors which had great influence on the extraction efficiency were selected as the independent variables of the response surface through the experimental design. The content of palmatine and berberine in *Coptis chinensis* was analyzed and optimized by response surface method.

## 3. Results and Analysis

### 3.1. HPLC Method Validation

**Figure 1:**
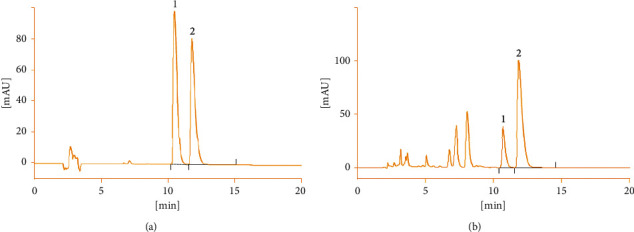
HPLC chromatogram (1, palmatine; 2, berberine).

The resolution of the chromatogram obtained by HPLC analysis of the mixed reference solution and *Coptis chinensis* extract was adequate. In addition, the number of theoretical plates was more than 3000 (Figure 1).

The mixed reference substance reserve solution was diluted 50, 25, 10, 5, 2.5, and 0 times, respectively. In addition, the peak area value was recorded by HPLC analysis. The calibration curve was drawn with peak area (*A*) as ordinate (*y*) and reference substance concentration (mg/mL) as abscissa (*x*). The regression equations of palmatine and berberine were obtained as *y* = 33054*x *−* *11.777 (*r* = 0.9997), the linear range was 0.011∼0.572 mg/mL; *y* = 36898*x *−* *352.3 (*r* = 0.9996) and the linear range was 0.023∼1.140 mg/mL, respectively.

The same extract of *Coptis chinensis* was injected continuously for 6 times according to the above HPLC analysis conditions. Besides, the RSD values of palmatine and berberine peak area were 1.45% and 1.33%, respectively, indicating that the instrumental precision was good. The RSD values of palmatine and berberine peak area calculated at 0, 2, 4, 6, 12, 24, and 48 h were 0.78% and 1.02%, respectively, according to the above HPLC analysis conditions, indicating that the solution was stable within 48 h.

Six samples of *Coptis chinensis* were analyzed (50 mg). In addition, 1.5 mL of DES-5 (water content 30%) was added, and *Coptis chinensis* was extracted according to the method described in Section 2.5. The average contents of palmatine and berberine were calculated to be 14.98 mg/g and 55.04 mg by HPLC analysis, and the RSD values were 1.77% and 1.62%, respectively. This indicated that the extraction method had good repeatability.

Six samples of Rhizoma Coptidis with known content, about 25 mg each, were precisely removed from palmatine reference solution (concentration 0.745 mg/mL) 0.5 mL and berberine reference solution (concentration 0.555 mg/mL) 2.5 mL in the same centrifuge tube. The well-proportioned *Coptis chinensis* powder was added, and DES-5 (water content 30%) 1.5 mL was added after steaming and drying the solvent in water bath. *Coptis chinensis* extract was extracted according to the method described in Section 2.5 and the extract was analyzed by HPLC. The average recoveries of palmatine and berberine were 100.1% and 98.7%, respectively, and the RSDs were 1.85% and 1.97%, respectively.

### 3.2. Effects of Different DESs, Molar Ratio, and Moisture Content of DES on the Contents of Palmatine and Berberine

**Figure 2:**
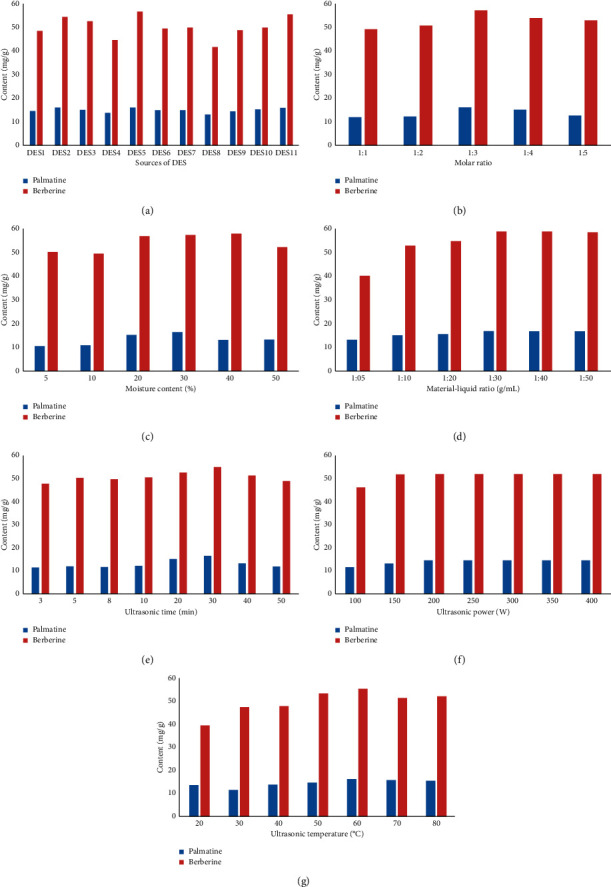
Effects of different types of DES (a), different ratios of DES (b), different moisture contents (c), different material/liquid ratios (d), different extraction times (e), different ultrasound powers (f), and different ultrasound temperatures (g) on the extraction rates of palmatine and berberine.

The composition of DESs determines its physical and chemical properties, which affects its extraction efficiency of natural compounds. The effects of 11 kinds of DESs (Table 1) on the contents of palmatine and berberine were studied. As shown in Figure 2(a), DES-5, the combination of choline chloride and phenol, was the most efficient in extracting palmatine and berberine from *Coptis chinensis*.

The viscosity of most DES decreases with the increase of temperature and the increase of hydrogen-bonded donors [11]. As can be seen from Figure 2(b), the extraction amount of palmatine and berberine from *Coptis chinensis* is the most when the molar ratio of choline chloride to phenol is 1 : 3. Less intramolecular hydrogen bonds may be formed, and the viscosity is very high when the ratio of choline chloride is low. It is not conducive to full contact with *Coptis chinensis* powder. The viscosity decreases and the extraction amount increases with the increase of phenol. The extraction amount of palmatine and berberine decreases when the molar ratio is more than 1 : 4. It may be caused by the instability of eutectic solvent and the decrease of hydrogen bond. Therefore, the molar ratio of 1 : 3 resulted more beneficial to the extraction of effective components of *Coptis chinensis*.

The addition of water reduces the viscosity of DESs. Moreover, the decrease of viscosity is beneficial to improve the cavitation effect of ultrasonic wave and reduce the loss of ultrasonic wave in the process of propagation [22]. Consequently, it is conducive to the extraction of berberine and palmatine in *Coptis chinensis* in a certain range. However, if we continue to increase the water content; it may affect the intermolecular hydrogen bond of DESs, change the structure of DESs, and reduce the solubility of effective components of *Coptis chinensis*. In this study, the effects of choline chloride-phenol eutectic solvents with different amounts of water on the extraction of palmatine and berberine from *Coptis chinensis* were investigated. As shown in Figure 2(c), the extraction amount of palmatine and berberine in *Coptis chinensis* also increased gradually with the increase of water content. However, it changed when the water content was more than 40%, and the extraction efficiency of *Coptis chinensis* decreased. This indicated that the water content of DES has a great influence on the extraction efficiency of *Coptis chinensis*.

### 3.3. Effect of Material-Liquid Ratio

The amount of solvent determines the efficiency to a certain extent of the whole extraction process. It can be seen from Figure 2(d) that the content of palmatine and berberine increases with the increase of material-liquid ratio. The extraction amount reached the highest point when the ratio of material to liquid was 1 : 30 g/mL. In addition, the contents of palmatine and berberine were almost unchanged when the ratio of material to liquid continued to increase. This may be because palmatine and berberine have been basically dissolved after the ratio of material to liquid is increased to a certain extent. If we simply increase the ratio of solvent, it has no obvious effect on the extraction of palmatine and berberine. Therefore, 1 : 30 g/mL was selected as the ratio of material to liquid for the extraction of palmatine and berberine from *Coptis chinensis*.

### 3.4. Effect of Ultrasonic Time, Ultrasonic Power, and Ultrasonic Temperature

Ultrasonication is an important step in the whole extraction process; sonication time directly affects the results. It can be concluded from Figure 2(e) that the extraction amount of palmatine and berberine from *Coptis chinensis* increases with the extension of time. The extraction efficiency of DESs reached the higher value with 30 min of sonication time. Despite this if time is too long, it may lead to the decomposition of active components of *Coptis chinensis* or other chemical reactions may occur. Consequently, the extraction value of palmatine and berberine decreases after 40 min.

Ultrasonic power also affects the extraction. Although ultrasonication can promote the dissolution of active substances, high ultrasonic power not only increases the experimental cost but also may cause changes in the structure of active substances. In the range of 100W to 400W, the content of palmatine and berberine in *Coptis chinensis* was the highest when the ultrasonic power reached 200W. Besides, more than 200W had almost no effect on the extraction effect, so the power of ultrasonic extraction was set at 200W (Figure 2(f)).

The appropriate extraction temperature is also an important parameter affecting the extraction of DES. Too high temperature may lead to the decomposition of the extract. Besides, if the temperature is too low, the extraction is not complete. It can be concluded from Figure 2(g) that the extraction content of palmatine and berberine in *Coptis chinensis* increased with the increase of temperature. In addition, it decreased when the extraction temperature exceeded 70°C. Moreover, it can be seen that increasing the temperature to 60°C can reduce the surface tension and viscosity of DES and make the extract easier to filter.

### 3.5. Response Surface Test Results and Analysis

According to the single-factor experimental results and Box-Behnken design principles, the test conditions are selected using the software Design-Expert 8.0.6. Moreover, the influencing factors such as moisture content (A/%), molar ratio (B), ultrasonic temperature (C/°C), and sonication time (D/min) were selected as independent variables, and palmatine (*Y*1, mg/g), berberine (*Y*2, mg/g), and total content (*Y*3, mg/g) were selected as evaluation indexes. The central point was tested in quintuplicate, the response surface analysis model was of 29 test sites with 4 factors and 3 levels. Response surface establishes optimum values for the control variables that will result in maximum activity or yield over the region evaluated in a specific experimental design [23]. The experimental design and response values are shown in Table 2, and the results of analysis of variance are presented in Table 3.

The quadratic polynomial equations of the response values *Y*1, *Y*2, and *Y*3 can be obtained by Design-Expert software and regression fitting of various factors.

 
*Y*1
  (mg/g) = 16.24 – 0.053A – 0.031 B + 0.016C –0.082C 0.0035D+0.026AB + 0.054AC – 0.042 AD – 0.034BC – 0.13BD – 0.011CD – 1.28A^2^ – 3.71B^2^ – 1.77C^2^ – 1.27D^2^ (*R*^2^ = 0.9982, *p<*0.001, lack of fit = 0.1849*>*0.05);
 
*Y*2
  (mg/g) = 57.31 + 0.0049A + 0.016B + 0.082C + 0.078D + 0.004AB – 0.0039AC+0.0073AD – 0.017BC – 0.026BD – 0.0083CD – 0.17A^2^ – 4.50B^2^ – 2.339C^2^ – 2.79D^2^ (*R*^2^ = 0.9889, *p<*0.001, lack of fit = 0.9995*>*0.05);
 
*Y*3
  (mg/g) = 73.55−0.048A – 0.015 B + 0.088 C + 0.084D + 0.031AB + 0.051AC – 0.034AD – 0.052BC – 0.16BD – 0.050CD – 1.46A^2^ – 8.21B^2^ – 4.15C^2^ – 4.05D^2^ (*R*^2^ = 0.9958, *p<*0.001, lack of fit = 0.9995*>*0.05).

According to the results, the three models were extremely significant (*p* < 0.001), and the misfit items were all >0.05, and the correlation coefficient *R*^2^ > 0.98. This indicated that the fitting degree of the model was high, and the equation could better reflect the relationship between the factors and the response surface.

**Figure 3:**
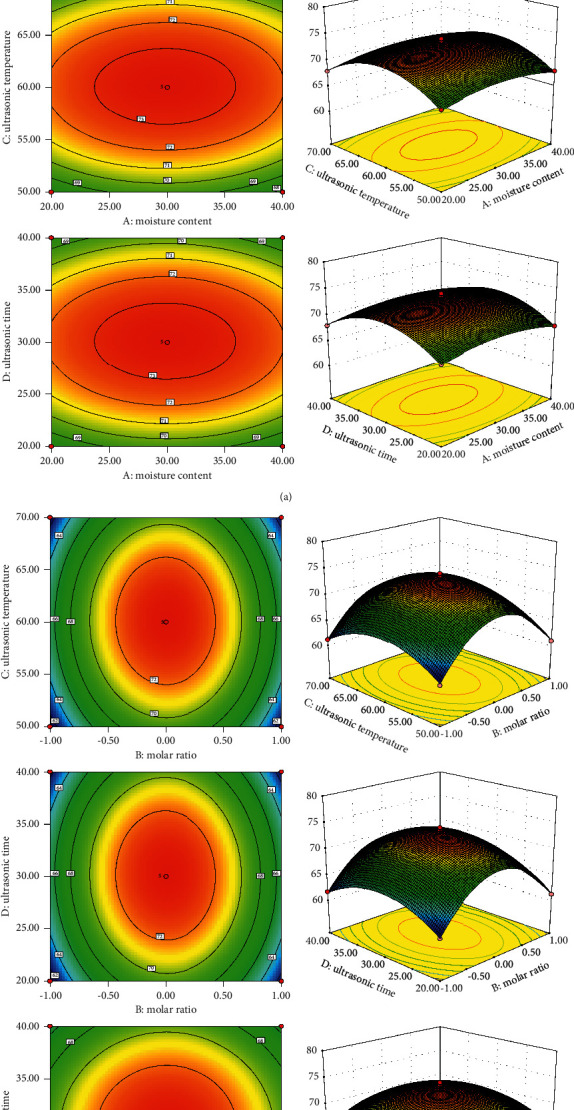
Response surface drawings of total content analyzed by interaction of two factors.

We can comprehensively evaluate the 3D response surface map and 2D contour map of the total content of palmatine and berberine and directly view the range of the best process parameters by using Design-Expert 8.0.6 software and according to the quadratic polynomial equation (Figure 3). As can be seen from the figure, the BD contours were oval, and the interaction is obvious.

The best process parameters can be obtained by solving the quadratic polynomial equation, and the mathematical model prediction of palmatine and berberine content can be calculated. It was predicted that the best extraction process parameters were as follows: water content was 29.83%, molar ratio was 1: 3, temperature was 60.10°C, and ultrasonic time was 30.09 min. Under this condition, the theoretical values of palmatine and berberine content were 16.2412 mg/g and 57.3066 mg/g, respectively. Combined with the actual needs, it was finally determined that the extraction process was as follows: water content was 30%, molar ratio was 1 : 3, temperature was 60°C, and ultrasonic time was 30 min. Three samples of *Coptis chinensis* were selected, and the contents of palmatine and berberine were calculated according to the optimum process parameters. The results showed that the average contents of palmatine and berberine were 16.7145 and 57.4013 mg/g, respectively. It was very close to the predicted value of the model. Therefore, the prediction of the model was accurate and reliable, and the best extraction process was desirable.

### 3.6. Comparison of Different Extraction Methods of *Coptis chinensis*

**Figure 4:**
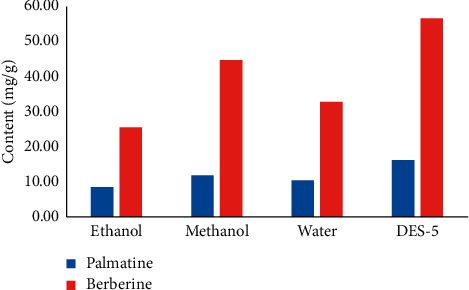
Comparison of different extraction methods.

Water and organic solvents are used as extraction solvents for the extraction of active compounds of traditional Chinese medicine at present. In addition, the main extraction methods are solvent extraction [24], ultrasonic extraction [25], and macroporous resin adsorption, among others [26]. Herein, an extraction process of ultrasound-assisted eutectic solvent was optimized. In addition, the results were compared with the extraction results of common extraction solvents such as water, methanol, and ethanol. It can be seen from the experimental process that this method has mild process conditions and does not need high temperature, ensures the stability of the extracted components, has a short extraction time, improves extraction efficiency, and saves time and cost [27, 28]. As can be seen in Figure 4, the extraction effect of the optimized ultrasound-assisted eutectic solvent extraction method was slightly better than those that use conventional extraction solvents. In addition, the low eutectic solvent is a green environmental protection natural solvent [29, 30].

## 4. Conclusion

In this study, *Coptis chinensis* was used as the research object, and low eutectic solvent system was constructed by using low eutectic solvent as the extractant, choline chloride as the hydrogen bond acceptor, and phenol as the hydrogen bond donor. The molar ratio of two eutectic solvents was 1 : 3, the water content of eutectic solvent system was 30% (v/v), and the ratio of material to liquid was 1 : 30 g/mL. Moreover, the extraction rate of berberine and palmatine was the highest with a sonication time of 30 min. The extraction rate of berberine and palmatine was affected by the ratio of material to liquid, temperature, and time in ultrasonic-assisted eutectic solvent extraction. DESs possess many preferable characteristics, including safety, nontoxicity, biodegradability, sustainability, low cost, and easy preparation, and it avoids the harm to the extraction personnel and environmental pollution.

The extract can be used in food, medicine, and other fields. It reflects the advantages of high yield and high speed of extracting active compounds by low eutectic solvent. This study not only provides a new method for the extraction of alkaloids from *Coptis chinensis* but also provides a reference for the extraction of active components of traditional Chinese medicine.

## Figures and Tables

**Table 1:** Sources of deep eutectic solvents.

Abbreviation	Hydrogen bond Receptor	Hydrogen bond Donor	Mole ratio
DES-1	Choline chloride	Maltose	1 : 1
DES-2	Choline chloride	Malic acid	1 : 1
DES-3	Choline chloride	Lactate	1 : 2
DES-4	Choline chloride	Fructose	1 : 1
DES-5	Choline chloride	Phenol	1 : 3
DES-6	Choline chloride	Glycerol	1 : 2
DES-7	Choline chloride	Propylene glycol	1 : 2
DES-8	Choline chloride	Xylitol	1 : 1
DES-9	Choline chloride	Urea	1 : 2
DES-10	Choline chloride	Acetic acid	1 : 2
DES-11	Choline chloride	Citric acid	1 : 2

**Table 2:** Design scheme and results of response surface test.

No.	Factor A	Factor B	Factor C	Factor D	Palmatine (mg/g)	Berberine (mg/g)	Total (mg/g)
1	30	1 : 3	60	30	16.3318	57.3551	73.6869
2	40	1 : 4	60	30	11.0627	52.7157	63.7784
3	30	1 : 2	50	30	10.6435	50.1267	60.7702
4	30	1 : 4	70	30	10.7245	50.6206	61.3451
5	20	1 : 3	60	40	13.7411	54.3042	68.0453
6	40	1 : 3	70	30	13.3213	54.7225	68.0438
7	40	1 : 2	60	30	11.1041	52.7099	63.814
8	30	1 : 3	60	30	16.2104	57.9285	74.1389
9	20	1 : 3	60	20	13.6131	54.3227	67.9358
10	30	1 : 3	60	30	16.1302	56.3247	72.4549
11	30	1 : 4	60	20	11.4241	49.8093	61.2334
12	30	1 : 2	60	40	11.5384	50.2042	61.7426
13	20	1 : 3	50	30	13.3432	54.6824	68.0256
14	30	1 : 3	50	40	13.1207	52.3281	65.4488
15	20	1 : 2	60	30	11.3957	52.7088	64.10449
16	30	1 : 2	70	30	10.7325	50.6117	61.3442
17	40	1 : 3	50	30	13.2233	54.6982	67.9215
18	30	1 : 3	60	30	16.2411	57.8209	74.0620
19	30	1 : 3	60	30	16.2903	57.0996	73.3899
20	30	1 : 3	70	20	13.2324	52.1982	65.4306
21	30	1 : 2	60	20	11.3214	49.6992	61.0206
22	20	1 : 3	70	30	13.2234	54.7222	67.9456
23	30	1 : 4	50	30	10.7732	50.2047	60.9779
24	30	1 : 3	50	20	13.1235	52.172	65.2955
25	30	1 : 4	60	40	11.1235	50.2113	61.3348
26	20	1 : 4	60	30	11.2514	52.6925	63.9439
27	40	1 : 3	60	20	13.6322	54.3141	67.9463
28	30	1 : 3	70	40	13.1861	52.1978	65.3839
29	40	1 : 3	60	40	13.5941	54.3248	67.9189

**Table 3:** Bivariate multiple regression fitting analysis of variance results of Box-Behnken experimental design.

Source	Sum of squares	df	Mean square	F value	*p* value
Model	517.23	14	36.94	234.88	<0.0001
A, moisture content	0.03	1	0.03	0.18	0.6805
B, molar ratio	0.00	1	0.00	0.02	0.8962
C, ultrasonic temperature	0.09	1	0.09	0.59	0.4558
D, ultrasonic time	0.09	1	0.09	0.54	0.4735
AB	0.00	1	0.00	0.02	0.8770
AC	0.01	1	0.01	0.07	0.8024
AD	0.00	1	0.00	0.03	0.8654
BC	0.01	1	0.01	0.07	0.7981
BD	0.10	1	0.10	0.61	0.4470
CD	0.01	1	0.01	0.06	0.8046
A^2^	13.83	1	13.83	87.90	<0.0001
B^2^	437.40	1	437.40	2780.84	<0.0001
C^2^	111.51	1	111.51	708.98	<0.0001
D^2^	106.18	1	106.18	675.03	<0.0001
Residual	2.20	14			
Lack of fit	0.35	10	0.01	0.74	0.9995
Pure error	1.85	4			
Cor total	519.43	28			

## Data Availability

The main table and figure data used to support the findings of this study are included within the article.
